# Strap associates with Csde1 and affects expression of select Csde1-bound transcripts

**DOI:** 10.1371/journal.pone.0201690

**Published:** 2018-08-23

**Authors:** Kat S. Moore, Nurcan Yagci, Floris van Alphen, Alexander B. Meijer, Peter A. C. ‘t Hoen, Marieke von Lindern

**Affiliations:** 1 Sanquin Research, Department of Hematopoiesis, and Landsteiner Laboratory Amsterdam UMC, University of Amsterdam, Amsterdam, The Netherlands; 2 Sanquin Research, Department of Research Facilities, Amsterdam, The Netherlands; 3 Department of Biomolecular Mass Spectrometry and Proteomics, Utrecht Institute for Pharmaceutical Sciences, Utrecht University, Utrecht, The Netherlands; 4 Centre for Molecular and Biomolecular Informatics, Radboud University Medical Center, Nijmegen, The Netherlands; Korea University, REPUBLIC OF KOREA

## Abstract

Erythropoiesis is regulated at many levels, including control of mRNA translation. Changing environmental conditions, such as hypoxia or the availability of nutrients and growth factors, require a rapid response enacted by the enhanced or repressed translation of existing transcripts. Cold shock domain protein e1 (Csde1/Unr) is an RNA-binding protein required for erythropoiesis and strongly upregulated in erythroblasts relative to other hematopoietic progenitors. The aim of this study is to identify the Csde1-containing protein complexes and investigate their role in post-transcriptional expression control of Csde1-bound transcripts. We show that Serine/Threonine kinase receptor-associated protein (Strap/Unrip), was the protein most strongly associated with Csde1 in erythroblasts. Strap is a WD40 protein involved in signaling and RNA splicing, but its role when associated with Csde1 is unknown. Reduced expression of Strap did not alter the pool of transcripts bound by Csde1. Instead, it altered the mRNA and/or protein expression of several Csde1-bound transcripts that encode for proteins essential for translational regulation during hypoxia, such as Hmbs, eIF4g3 and Pabpc4. Also affected by Strap knockdown were Vim, a Gata-1 target crucial for erythrocyte enucleation, and Elavl1, which stabilizes *Gata-1* mRNA. The major cellular processes affected by both Csde1 and Strap were ribosome function and cell cycle control.

## Introduction

Maintenance of correct numbers of erythrocytes in peripheral blood requires continuous replenishment with newly synthesized cells. Proliferation and differentiation of erythroblasts needs to be tightly balanced to prevent anemia and ischemic damage of organs, or an excess of erythrocytes and a risk for stroke. Environmental factors such as growth factors (e.g. erythropoietin and stem cell factor) or nutrients (e.g. iron) are crucial to control erythropoiesis, which occurs in part through control of translation of the available transcriptome. RNA binding factors have an important role in control of translation. For instance, iron regulatory proteins 1 and -2 (Irp1, Irp2) bind to the iron response element in *Ferritin* and *Transferrin receptor* mRNA to control expression of the encoded proteins that are crucial to erythropoiesis [1]. Zinc finger binding proteins 36 like 1 and -2 (Zfp36l1, Zfp36l2) bind to a large number of transcripts and deletion of Zfp36l2 disrupts erythropoiesis [2,3]. The RNA-binding protein Csde1 (cold shock domain protein e1), first described as Unr (upstream of Nras) [4], is widely expressed, but expression levels differ per cell type. In the hematopoietic system, expression of Csde1 is increased more than 100-fold in erythroblasts relative to other hematopoietic cells, and expression of Csde1 is reduced in the congenital anemia Diamond Blackfan Anemia (DBA), characterized by haploinsufficiency of ribosomal proteins involved in ribosome biogenesis [5]. Knockdown of Csde1 impairs both proliferation and differentiation of erythroblasts [5].

Csde1 regulates the fate of target transcripts by binding to the 3’ UTR [4,6] or to IRESs (Internal Ribosomal Entry Sites) [7–10]. Because Csde1 is capable of binding a broad variety of mRNAs containing A/G-rich binding motifs, it is likely that it functions as a global regulator of translation [11,12]. By consequence, it is involved in diverse processes, including X-chromosome dosage compensation in Drosophila [13], cell cycle control [10], and control of metastasis in melanoma [14]. We recently identified the transcripts bound by Csde1 in erythroblasts [15]. These transcripts encoded proteins involved in protein homeostasis: translation factors, ribosome biogenesis factors, subunits of the proteasome and peptidases.

The function of RNA-binding proteins depends on associated proteins. For instance, RNA binding proteins that interact with AU-rich elements in the 3’UTR of transcripts such as Auf1 (AU-rich element binding factor) can interact either with the pre-initiation scanning complex to enhance translation, or with the Cnot1 (Ccr4/Not complex 1) complex which results in deadenylation [16]. Similarly, the role of Csde1 is likely influenced by associated proteins that may affect its RNA-binding affinity and/or functional consequences. Csde1 cooperates with Pabp (PolyA binding protein) when interacting with the 3’ UTR [17,18] and with PTB (polypyrimidine tract binding protein) and hnRNP (heterogeneous nuclear binding protein) C1/C2 when interacting with internal ribosomal entry sites (IRESs) [7,10,19]. It also interacts with Strap (serine-threonine receptor associated-protein, also called Unrip) [20]. Strap is a member of a large family of WD40 (Trp/Asp) repeat-containing proteins that are known to function as relatively promiscuous adapters. WD40 domains function as a platform for protein/protein interactions. The association of WD40 domains with phosphorylated Ser-Thr residues often place WD40 domain proteins in network nodes of signaling cascades [21]. Strap is involved in numerous biological pathways, including TGFβ signaling [22,23], MAPK signaling [24], Wnt signaling [25], Notch signaling [26] and assembly of the survival motor neuron (SMN) complex [27]. Its association with the SMN complex is mutually exclusive with Csde1 binding. Strap also associates with 4E-T (eIF4E Transporter), together with Csde1 [28]. Taken together, however, little is known about the function of Strap when bound to Csde1.

The aim of this study was to investigate Csde1 protein complex formation in erythroblasts. Strap was the most strongly associated protein in mouse erythroblasts. Strap knockdown did not alter transcript binding by Csde1, but reduced protein expression of many Csde1-bound transcripts and enhanced expression of some other transcripts. Proteins whose expression was regulated by Strap are involved in terminal erythroid differentiation, ribosome biogenesis, cell cycle regulation, and the hypoxic response.

## Results

### Strap strongly associates with Csde1

To investigate which proteins form a complex with Csde1 in erythroblasts, we utilized the strong affinity of streptavidin-biotin interaction as an efficient alternative to antibody-based immunoprecipitation to purify Csde1 protein/mRNA complexes [29,30]. BirA and biotagged Csde1 were coexpressed in Mel cells [5]. Csde1-bound protein complexes were enriched on streptavidin-coated Dynabeads under conditions that preserve binding of target mRNAs [15]. Mel cells expressing the biotin ligase BirA without biotagged Csde1 were used as a control.

**Fig 1:**
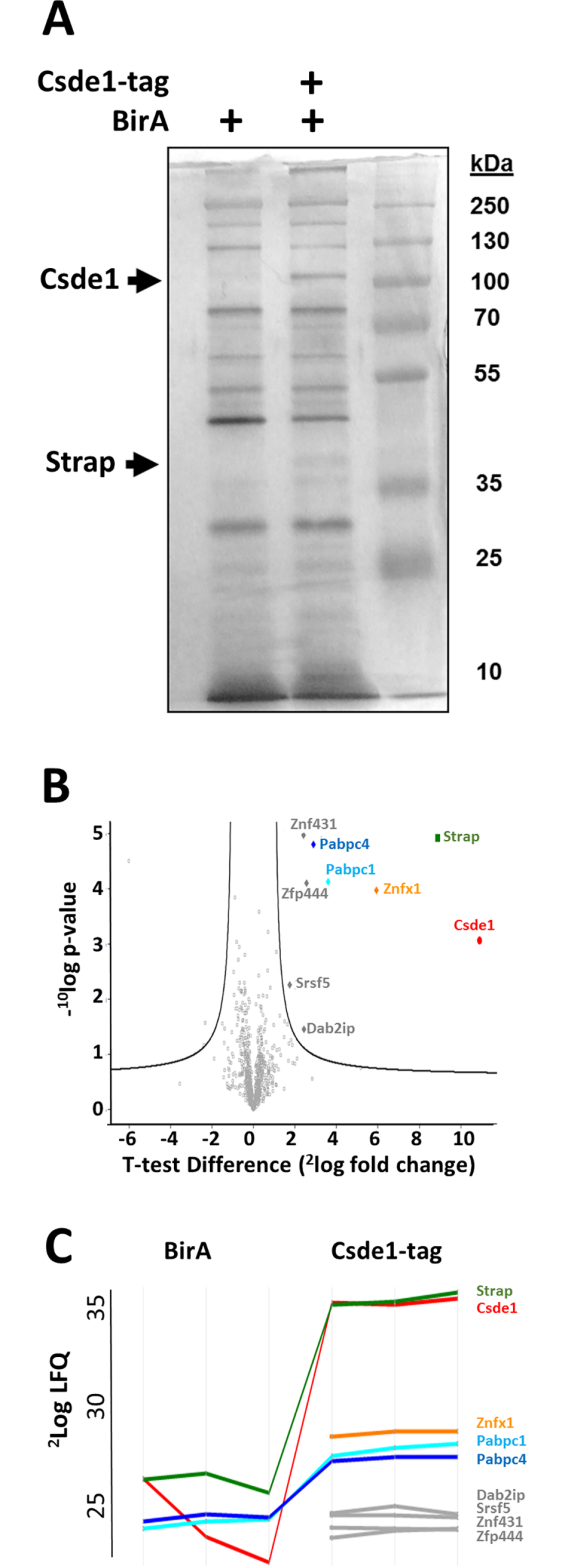
Proteins associated with Csde1 upon pull down of biotagged Csde1. **(A)** Lysate of MEL cells expressing the biotin ligase BirA with or without biotagged Csde1 was incubated with streptavidin beads, washed, and eluted in laemmli buffer before loading on SDS-PAGE and silver staining. Third lane is a protein size ladder, sizes indicated in kD next to the lane. **(B)** Mass spectrometry of proteins pulled down by Csde1, and analysis by two-way t-test revealed 8 Csde1-associated proteins at a significance threshold of S0 = 0.8. -10log p-value is plotted against 2log fold change (n = 3). **(C)** Protein profile expression plot of Csde1-associated proteins (LFQ: Label free quantification). Lines are discontinued when peptides were not detected in control pull down in BirA only MEL cells.

SDS-PAGE of cell lysates and subsequent silver staining showed a series of bands representing endogenously biotinylated proteins that are common to both the Csde1-pulldown lane and the BirA pulldown control (**Fig 1A**). Selectively present in the Csde1-pulldown lane were two bands at ~100kDa and ~45kDa, representing Csde1 itself and an unknown associated protein, respectively. Mass spectrometry analysis of the bio-Csde1 pulldown eluate and the BirA pulldown control eluate identified proteins enriched in the Csde1 fraction. A two-way *t*-test, applying an artificial within groups variance (S0) [31] of 0.8, was used to set the threshold for significant binding (**Fig 1B, Table 1**). These proteins included Znfx1 (Zinc finger NFx1-type containing 1), Pabpc1 and Pabpc4. Notable is the high enrichment of Strap (**Fig 1C**). Strap was nearly twice as abundantly associated with Csde1 in erythroblasts than the next highest enriched protein (Znfx1, predicted mass 220kDa). Strap was previously shown to associate with Csde1/Unr [20], but little is known about how the two proteins cooperate to affect Csde1’s function.

**Table 1:** Significant proteins from MS Csde1 pulldown vs BirA.

	LFQ (2log)					
	BirA^$^		Csde1-tag			
Gene names	Av (n = 3)	St. Dev	Av (n = 3)	St. Dev	P-value^&^ -^10^log	T-value
Strap	26.27	0.49	35.15	0.32	4.92	7.82
Csde1	24.12	2.11	35.02	0.15	3.06	5.40
Znfx1	NaN	NaN	28.63	0.14	3.97	4.99
Pabpc1	24.25	0.23	27.85	0.29	4.13	3.54
Pabpc4	24.51	0.16	27.41	0.12	4.81	3.16
Znf431	NaN	NaN	23.97	0.04	4.97	2.73
Zfp444	NaN	NaN	23.79	0.23	4.10	2.69
Srsf5	NaN	NaN	24.60	0.05	2.26	1.56
Dab2ip	NaN	NaN	24.81	0.22	1.46	1.54

$ NaN = Not detected

& Two-way t-test at S0 threshold S0 = 0.8

### Loss of Strap does not affect Csde1’s ability to bind transcripts

**Fig 2:**
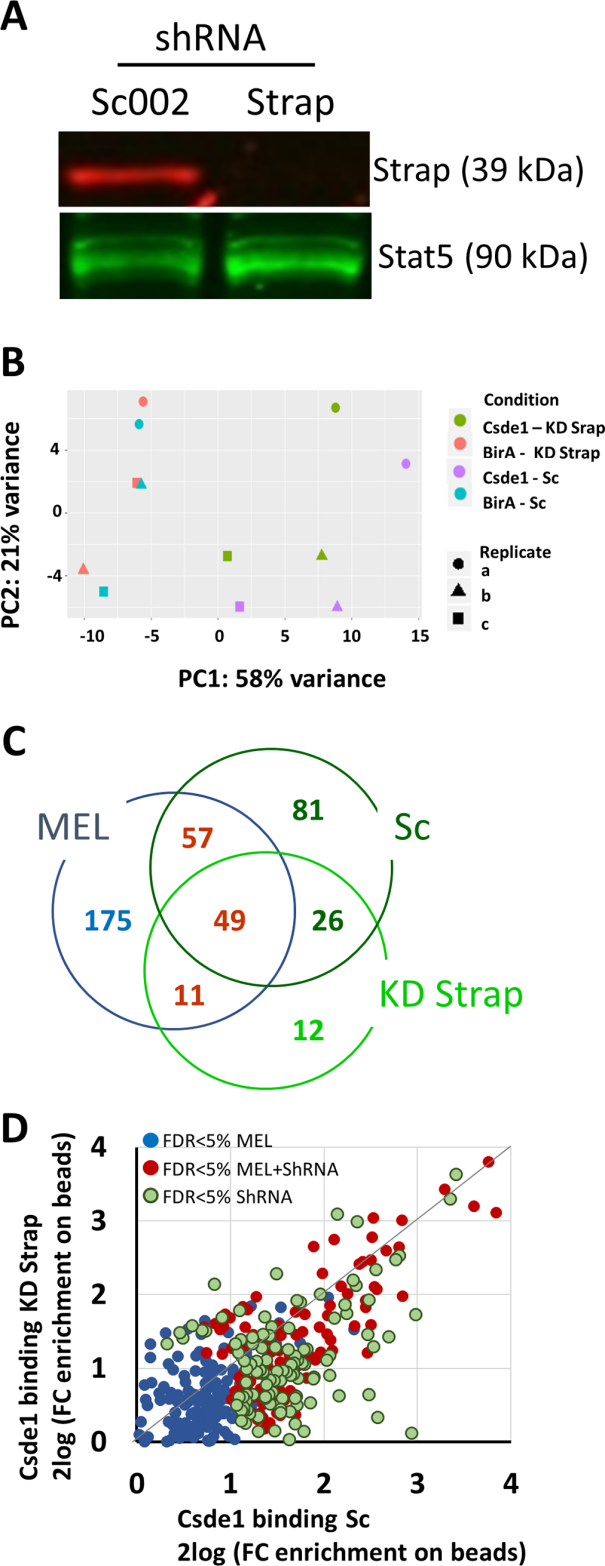
Knockdown of Strap does not affect the pool of Csde1-bound transcripts. **(A)** MEL cells expressing BirA and biotagged Csde1 were treated with a short hairpin targeting Strap or a non-targeting short hairpin (Sc). Knockdown of Strap was confirmed by Western blot staining for Strap and Stat5 as a loading control. **(B)** Principal component analysis of transcripts bound to streptavidin beads incubated with MEL cells expressing BirA with or without biotagged-Csde1 and treated with anti-Strap shRNA (KD Strap) or control shRNA (Sc). Three biologically independent experiments were performed (a-c) **(C)** Venn diagram showing the overlap between Csde1-bound transcripts detected at a FDR<0.05 (biotag-Csde1 plus BirA versus BirA only) in previous experiments in MEL cells [15] and in MEL cells expressing control shRNA (Sc) or shRNA against Strap (KD Strap) (**D**) The enrichment of transcripts on streptavidin beads (biotag-Csde1 + BirA versus BirA only) compared between MEL cells expressing control shRNA (Sc) or shRNA against Strap (KD Strap) plotted for the combined transcripts as shown in (C). (C/D) red numbers/dots detected in parental MEL and in shRNA treated MEL; blue numbers/dots detected in previous study with parental MEL; green numbers/dots detected in shRNA treated MEL.

We previously identified 292 transcripts that were enriched upon pull down of biotagged Csde1 from MEL cells with a Benjamini-Hochberg false discovery rate (FDR) cutoff of 5% [15]. To investigate whether association with Strap is required for the binding of transcripts by Csde1, Strap expression was reduced in BirA and BirA/biotag-Csde1 expressing MEL cells using a Strap targeted shRNA expressed transduction and a non-targeting control short hairpin (Sc). Knockdown of Strap was confirmed by Western blotting (**Fig 2A**). Next, we pulled down biotagged Csde1 from MEL cells expressing BirA only, or BirA plus biotagged Csde1 to identify Csde1 protein complexes in presence and absence of Strap. RNA was isolated from the Csde1 protein complexes and subjected to high-throughput RNA sequencing. Principal component analysis (**Fig 2B**) on previously identified Csde1-bound transcripts [15] showed that the presence of biotagged Csde1 in presence or absence of Strap counts for the majority of variance (PC1, 58%), representing the effect of the pulldown versus the control (BirA). In PC2 (21%), Csde1 pulldown samples clustered by replicate, not by the abundance of Strap.

We first identified Csde1-associated transcripts in presence and absence of Strap upon transduction of MEL cells with shRNA expression vectors. In Strap knockdown cells, a total of 98 transcripts were significantly enriched (Wald test with FDR < 0.05) after Csde1 pulldown, compared to 213 in cells treated with control Sc shRNA (**Fig 2C, S1 Table**). The abundance of transcripts associated with Csde1 in the pull down eluate from MEL cells that do or do not express Strap seemed comparable (**S1 Fig, panel A**). Targets that were significantly enriched in one pulldown were often just below the significance threshold in another. Approximately half of the Csde1-bound transcripts detected in shRNA treated MEL cells overlapped with previously identified Csde1-bound transcripts in parental MEL cells (**Fig 2C, S1 Table**) [15]. Viral transduction affects gene expression, including Csde1, which may explain differences in detection levels of Csde1-bound transcripts [15]. Indeed, the detection of transcripts in pull down eluates of SC shRNA treated and non/treated MEL WT cells differed (**S1 Fig, panel B**). The enrichment of transcripts in pulldowns from BirA/biotagged Csde1 versus BirA MEL cells seemed comparable in cells with or without reduced Strap expression (**Fig 2D, S1 Fig**).

Because certain transcripts may fall just above or below a significance threshold across multiple conditions, a Venn diagram-style comparison is potentially misleading. Addressing this issue requires the application of a mathematical model capable of quantifying the combined effect of multiple conditions (pulldown vs background *and* KD Strap vs Sc) on individual transcripts. In regression analysis, this is accomplished by the use of an interaction term. To determine whether Strap expression significantly alters the binding of some transcripts by Csde1, we used an interaction model that considers both the likelihood that a transcript is significant in the pulldown and the likelihood that Strap knockdown is affecting the outcome. This model indicated that Strap does not significantly alter Csde1 transcript binding. Although the interaction model identified 31 transcripts at an FDR cutoff of 5% (**S2 Table, S2 Fig**), these transcripts were exclusively present in very low abundance across all samples. Importantly, none of the transcripts identified in the interaction model were identified Csde1-bound transcripts (they were not selectively precipitated from lysates containing biotagged Csde1).

For further analysis, we defined the population of Csde1-bound transcripts as the sum of transcripts detected either in untreated MEL, in MEL expressing Sc, or anti-Strap shRNA. Because this list is larger than we previously published for MEL cells, we reanalyzed transcripts for enriched pathways driven by encoded proteins using Genetrail2 [32]. At an FDR significance cutoff of 0.05 were biological process GO terms for: translation (initiation), (m/r)RNA processing, splicing, cell division, and mitochondrial organization, as well as KEGG pathway terms for ribosome, proteasome, and RNA transport (among others) (**S3 Table**).

### Strap controls transcript and protein expression of select Csde1-bound transcripts

**Fig 3:**
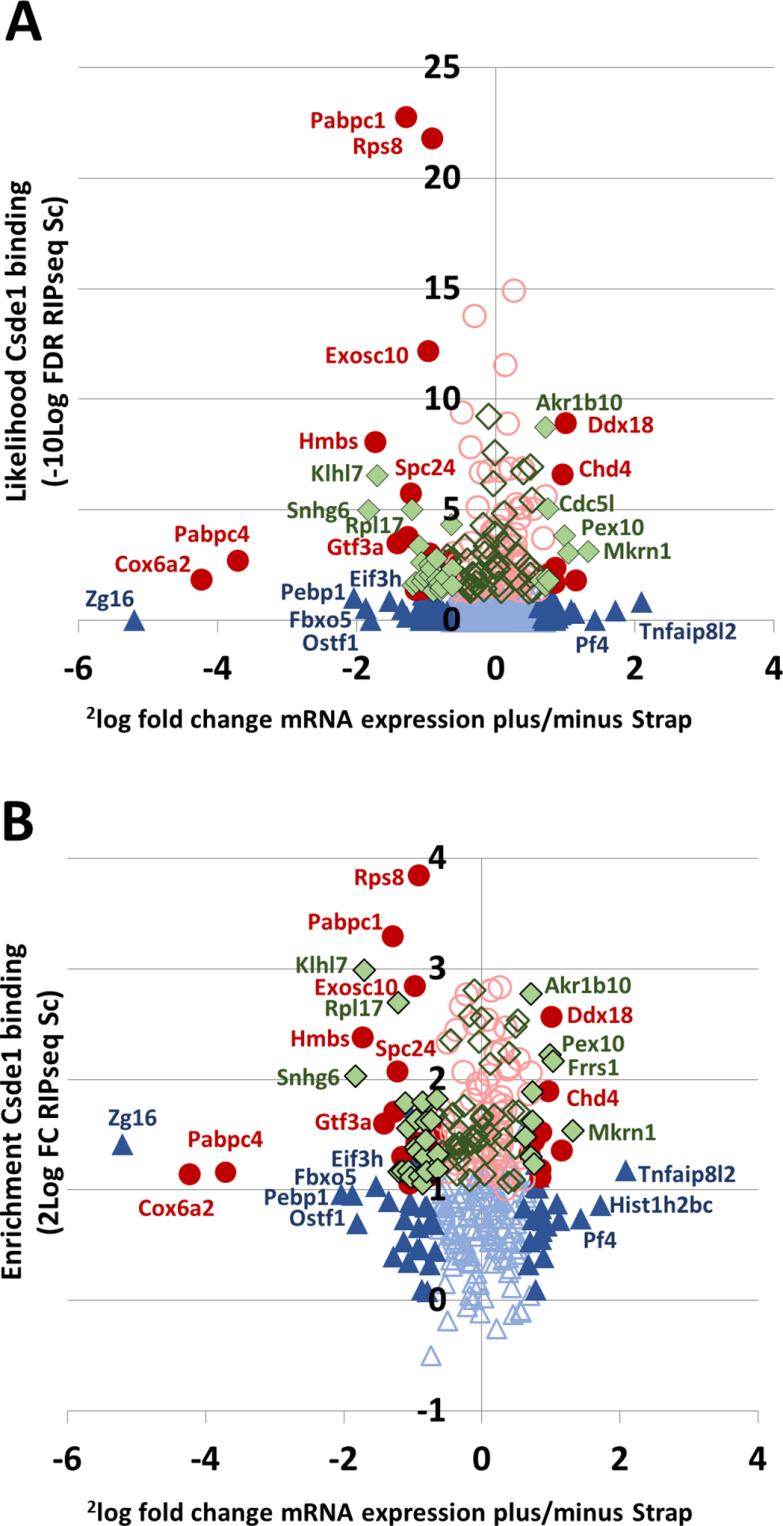
Strap knockdown affects the mRNA expression of select Csde1-bound transcripts. MEL cells were treated with control and anti-Strap shRNA in 3 independent experiments. Cells were processed for transcriptome analysis by RNA sequencing. **(A/B)** The fold change expression of transcripts in Strap shRNA versus Sc shRNA treated MEL cells (2log values; X-axes) was compared to the likelihood of that transcript being bound to Csde1 in Sc treated MEL cells (likelihood enrichment on streptavidin beads in Sc treated MEL cells expressing biotagged Csde1 plus BirA compared to the SC treated MEL cells expressing BirA only; -10log values; Y axes) (**A**), or to the fold-change enrichment of that transcripts on streptavidin beads incubated with lysate of Sc treated MEL cells (**B**). All transcripts selected as in Fig 2C and 2D are included. Transcripts indicated by closed symbols are differentially expressed in Sc and anti-Strap treated MEL cells at FDR<0.05. Blue triangles represent transcripts previously reported as Csde1-bound transcripts in MEL cells with FDR <0.05; green diamonds are transcripts only associated with Csde1 at FDR<0.05 in shRNA treated MEL cells; red circles were detected at a FDR<0.05 in both shRNA treated and non-treated MEL cells.

Strap may affect Csde1 function with respect to mRNA stability and translation. We performed RNA sequencing on total mRNA, and mass spectrometry on cell lysates, after targeting Strap for shRNA-mediated knockdown in MEL cells, using a control shRNA (Sc) to control for the effects of viral exposure. Principal component analysis of RNA sequencing results showed that the majority of variation (87%) can be explained by the knockdown of Strap (**S3 Fig**). After Strap knockdown, 3828 transcripts were differentially expressed with a FDR < 0.05, of which 102 represented Csde1-associated transcripts (**S4 Table**). Thirty-Seven Csde1-bound transcripts increased in expression while 65 Csde1-bound transcripts decreased in expression in response to Strap knockdown. The observed change in expression was not related to the likelihood of being a Csde1 bound transcript (**Fig 3A**), nor by the enrichment of a transcript in Csde1 pull down (**Fig 3B**). Csde1-bound transcripts altered by Strap knockdown included transcripts encoding several translation factors (*Eif1*, *Eif2B1*, and *Eif3H*, *Pabpc1*, *Pabpc4*), proteasome subunits (*Psme1*, *Psmc1*, *Psma2*), RNA processing/transport (*Exosc1/Exosc10*, *Thoc5)* and proteins involved in cell cycle control (*Fbxo5*, *Spc24*, *Kif23*), often via (de)ubiquitinylation (*Actr8*, *Ube2c*, *Cdc23*). Also notable were *DEAD-domain protein X18* (*Ddx18*), *Ran-binding protein 1 (Ranbp1)*, *hydroxymethylbilane synthase (Hmbs*, involved in heme biosynthesis), *Platelet factor 4 (Pf4)* and *osteoclast stimulating factor 1 (Ostf1)* (**S4 Table**).

**Fig 4:**
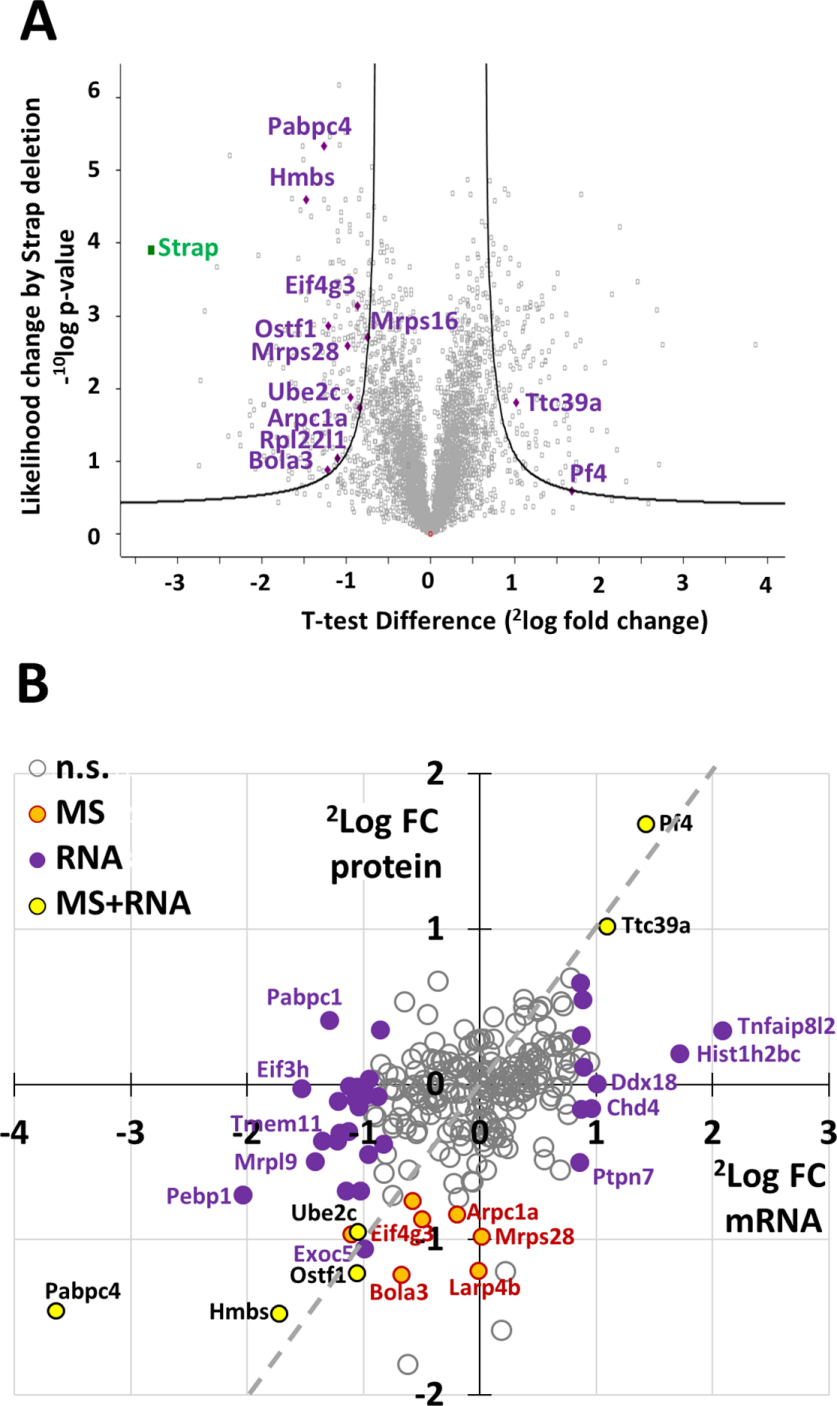
Strap knockdown affects the protein expression of select Csde1-bound transcripts. MEL cells were treated with control and anti-Strap shRNA in 3 independent experiments. Cell lysates were analyzed by mass spectrometry. **(A)** The fold change (Strap KD vs Sc) expression of all detected proteins in MEL cells (2log values; X-axes) was plotted against the likelihood of the protein being differentially expressed (-10log values; Y-axes). Grey lines indicate a threshold of S0 = 0.8. Highlighted are all proteins that are differentially expressed above threshold encoded by Csde1-bound transcripts. (**B**) Effect of Strap knockdown on Csde1-bound transcripts at RNA and protein level. On the Y axis, 2log fold change (Strap KD vs Sc) in average protein expression (iBAQ) plotted against 2log fold changes (Strap KD vs Sc) in average RNA expression (RPKM) on the X axis, for Csde1-bound transcripts only. purple circles: differential RNA (not protein) expression at FDR<0.05, orange circles: differential protein (not RNA) expression at FDR<0.05; yellow circles: both RNA and protein differentially expressed. The grey striped line indicates similar change in protein and transcript expression.

In parallel, we analyzed protein expression in Mel cells transduced with lentiviral vectors expressing shRNA against Strap or Sc control shRNA. We used label-free quantification (LFQ) of mass spectrometry to compare protein expression in total cell lysates and analyzed LFQ values with a two-way *t*-test, which identified 404 proteins as differentially regulated after Strap knockdown with an artificial within groups variance (S0) cutoff of 0.8 (**S5 Table**). Thirteen of the differentially regulated proteins after Strap knockdown were encoded by Csde1-bound transcripts (**Table 2**; **Fig 4A**). The lower number of differentially expressed proteins, compared to differentially expressed transcripts, may partly be technical. Proteins expressed at lower levels are not reliably measured, whereas mRNA was measured at greater depth. Yet, the RNA-binding Csde1 complex may differentially control mRNA translation, causing discrepancies between mRNA and protein expression levels. For some differentially expressed proteins, the transcript was not affected by Strap knockdown, while in other cases, both transcript and protein expression were affected by Strap knockdown.

**Table 2:** Differentially expressed proteins encoded by Csde1-bound transcripts upon Strap KD.

				LFQ (2log)			
				Sc		KD Strap	
Gene names	Function	P-value -^10^log	T-value	Average (n = 3)	St. Dev	Average (n = 3)	St. Dev
Pabpc4	Regulation of mRNA stability	5.34	-1.50	32.34	0.05	31.09	0.05
Hmbs	Heme biosynthesis	4.60	-1.70	32.29	0.07	30.82	0.09
Eif4g3	Translational initiation	3.14	-0.97	29.41	0.08	28.54	0.14
Ostf1	Osteoclast formation & bone resorption	2.86	-1.27	30.56	0.15	29.34	0.22
Mrps15	Mitochondrial translation	2.70	-0.83	27.71	0.18	26.96	0.03
Mrps28	Mitochondrial ribosomal protein	2.59	-1.04	28.02	0.09	27.04	0.24
Larp4B	La-related protein 4B	2.08	-1.14	29.51	0.11	28.31	0.41
Ube2c	Ubiquitinylation, cell cycle	1.88	-0.93	30.08	0.20	29.13	0.33
Ttc39a	Unknown	1.81	0.97	NA	NA	26.33	0.31
Arpc1a	Actin filament polymerization	1.73	-0.82	28.12	0.23	27.29	0.30
Rpl22l1	Ribosomal protein	1.16	-0.81	29.84	0.12	28.88	0.67
Bola3 *	Respiratory chain complex assembly	0.87	-0.84	27.43	0.87	26.20	0.72
Pf4 **	Chemotaxis, platelet aggregation	0.59	0.81	29.29	0.05	29.72	0.41

NA: not detected

* Peptide not detected in one of three KD Strap samples (n = 2)

** Peptide not detected in one of three Sc samples (n = 2)

To establish the role of posttranscriptional control, we plotted the fold-change (Strap knockdown versus Sc control) in protein (average iBAQ) and transcript (average RPKM) expression for all Csde1-associated transcripts (**Fig 4B**). This demonstrated that the presence of Strap had distinct effects on expression of mRNA and protein of Csde1-bound transcripts. Expression of mRNA may be reduced, which is compensated by increased protein expression (e.g. Pabpc1) or mRNA expression can be increased whereas protein expression is decreased (e.g. Ptpn7, protein tyrosine phosphatase N7). In both cases, this results in differential mRNA expression, but no statistically significant change in protein expression. Among the downregulated proteins are Pabpc4, a protein that we also found to be associated with Csde1, and which is essential for terminal erythroid differentiation [33]. Pabp proteins directly bind the Eif4g scaffold protein in the Eif4f cap-binding complex to enhance translation [34]. This complex also binds Eif3, of which the Eif3h peptide is encoded by a Csde1-bound transcript controlled by Strap. Eif4g3 protein expression is reduced at low Strap levels. It is a variant scaffold protein involved in translation of a select set of transcripts under hypoxic conditions [35]. Interestingly, some Csde1-bound transcripts regulated by Strap are transcriptionally controlled by hypoxia, such as heme biosynthesis protein hydroxymethylbilane synthase (Hmbs) and osteoclast stimulating factor 1 (Ostf1) [36,37]. Thus, GO-term analysis suggests Strap and Csde1 may coordinate mRNA translation and cell cycle divisions, and may also be involved in the hypoxic response in erythroblasts.

### Role of Strap in expression of Csde1-bound versus non-Csde1 associated transcripts

**Fig 5:**
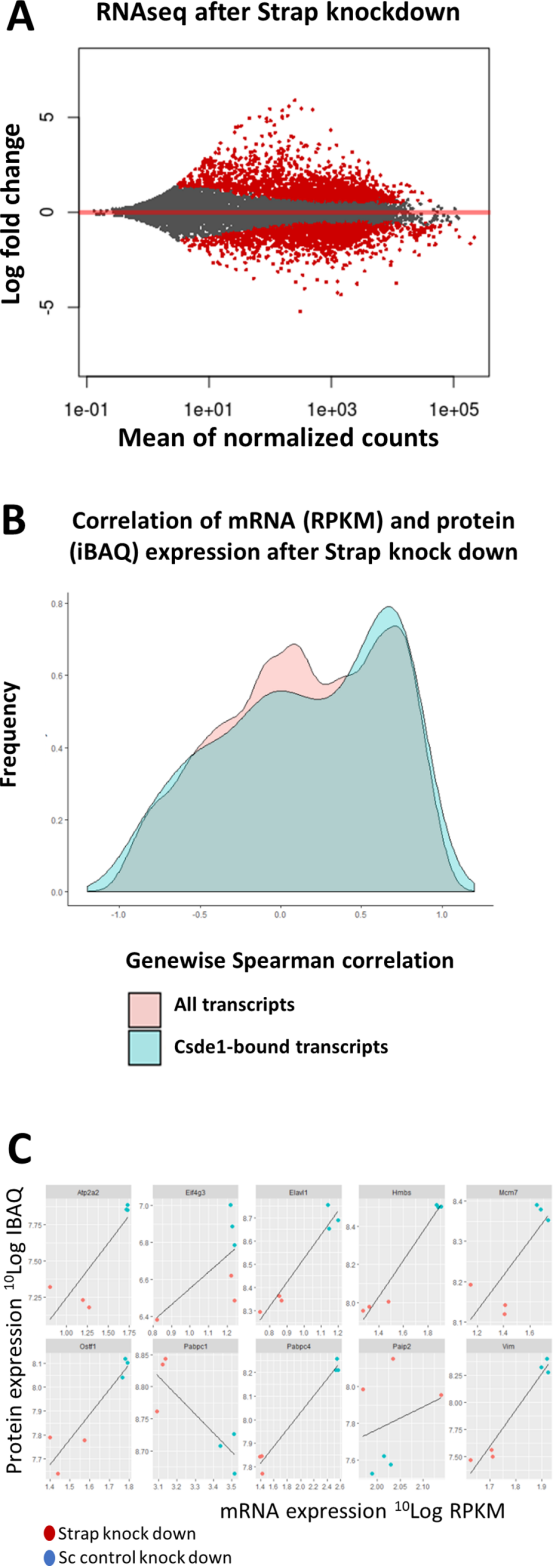
Total transcript and protein expression controlled by Strap. **(A)** MA plot depicting the fold change of mRNA expression (2log cpm) in MEL cells with or without Strap knockdown, plotted against the average expression of the specific mRNA (10log cpm). Transcripts with a FDR<0.05 are highlighted in red (n = 3). **(B)** Density plot of genewise Spearman rank correlation coefficients in MEL cells treated with anti-Strap and control shRNA. A comparison of Csde1-bound transcripts (blue) versus all transcripts detectable by both RNAseq and mass spectrometry (red) shows no significant different in the correlation RNA-protein expression. **(C)** Correlation between transcript (10Log RPKM) and protein (10log iBAQ) expression levels of selected Csde1-bound transcripts.

In addition to the regulation of Csde1-associated transcripts, Strap knockdown affected the expression of a large number of genes (**Fig 5A**). Gene set enrichment analysis (GSEA) on the differentially expressed transcripts upon Strap KD may give an indication of the Csde1 independent role of Strap. For this, we used Genetrail2 [32]. Of particular interest is the number of biological process GO terms related to cell cycle and ribosomal KEGG pathways, which overlap with pathways enriched among Csde1-bound transcripts. It is notable that known Strap pathways such as TGF-B and MAPK were not enriched (**S6 Table**). Because mass spectrometry is less powerful in detecting proteins that are expressed at low levels, it is not surprising that we detected fewer differentially expressed proteins. Overrepresentation analysis (ORA) on proteins differentially expressed beyond a threshold of S0 = 0.8 revealed a diverse array of cellular functions, with biological process GO terms related to cell division, metabolic processes, and vacuole organization of particular prominence within the enriched terms (**S7 Table**). KEGG pathway enrichments include endocytosis, lysosome/peroxisome functionality in addition to several viral-response pathways. The latter can be seen as a predictable consequence to shRNA introduction via viral constructs.

To assess whether Strap knockdown affects the ratio between RNA and protein levels, we calculated both sample-wise and gene-wise Spearman correlation coefficients between RNA and protein. Comparisons were limited to genes with at least one valid value within both the RNA sequencing and mass spectrometry datasets. An additional correlation analysis was performed on previously identified Csde1-bound transcripts, exclusively. RNA (RPKM) and protein (iBAQ) expression was 10log-transformed prior to calculating the correlation coefficient. Strap knockdown did not induce significant changes in global correlations in mRNA and protein expression (**Table 3**). The lack of discernable differences was maintained between all observable genes and previously identified Csde1-bound transcripts specifically. A similar lack of differences is evident in a genewise comparison between all observable genes and Csde1-bound transcripts (**Fig 5B**). Striking differences were observed for gene specific correlations between RNA and protein (**S9 Table**). Pabpc1 protein and RNA expression were negatively correlated with Strap expression in MEL cells expressing anti-Strap or control shRNA, paradoxically indicating a higher protein expression at lower levels of mRNA (**Fig 5C**). Interestingly, Strap knockdown reduced mRNA and protein expression of Pabpc4 while increasing expression of its antagonist Paip2, (poly(A) binding protein interacting protein 2). Vim, Atp2a2, Elavl1, Pf4, Ostf1, Hmbs and Mcm7, all of which are implicated in the hypoxic response, are significantly altered in expression at both RNA and the protein level in response to Strap knockdown. Thus, regulation seems to occur mainly at the transcriptional level. By contrast, protein expression of Eif4g3 was strongly reduced by Strap knockdown while RNA expression remains constant, suggesting that Eif4g3 is regulated by Strap at the translational or posttranslational level.

**Table 3:** Sample-wise correlations between RPKM and iBAQ after Strap knockdown.

	Spearman correlation coefficient	
Sample	All genes	Csde1 targets
KD Strap a	0.51	0.53
KD Strap b	0.53	0.52
KD Strap c	0.50	0.53
Sc a	0.51	0.53
Sc b	0.52	0.55
Sc c	0.52	0.56

### Additional roles of Strap

The association of Strap with Csde1 was shown to be mutually exclusive with binding to survival motor neuron (SMN) complex protein Gemin7 [27], suggesting that Csde1 may compete with other pathways for binding to Strap. Immunoprecipitation of Csde1 suggests that Csde1 does not completely sequester Strap, and cell fractionation experiments indicate that part of Strap, but not Csde1, localizes to the nucleus (**S4 Fig, panel A**). To investigate whether Csde1 expression could alter the distribution of Strap, we tested subcellular distribution of Strap in MEL cells with a Crispr/Cas-induced in-frame deletion (Hypomorph) in Csde1, in which Csde1 expression was less than 50% of WT MEL levels, or with an out-of-frame deletion (hom KO) [15]. The nuclear fraction of Strap was not increased in Csde1 mutant clones, indicating that Csde1 does not sequester Strap from the nucleus (**S4 Fig, panel B**).

Expression of shRNA targeting Strap in these Csde1 deficient cells would discriminate Csde1 dependent and independent effects of Strap. However, the Csde1 Del clones expressed low but significant levels of truncated Csde1 protein. The inability to obtain cells that completely lack Csde1, and the selection for survival in Csde1-deficient clones complicated expression analysis in these cells [15].

Through binding of SMN protein Gemin7 Strap, but not Csde1, may be associated with mRNA splicing [27]. Therefore, the RNAseq data were analyzed using DEXSeq to detect differential expression at the exon level (**S9 Table**) [38,39]. The analysis detected alternative expression of single exons in 7% of all Csde1-bound targets (30 exons in 26 transcripts), and in 8% of differentially expressed transcripts (545 exons in 315 transcripts; **S5 Fig, panel A**). Because more genes show differential expression of their transcripts compared to differential expression of individual exons, we conclude that the presence of Strap mainly controls differential expression at the transcriptional level. To detect alternative splicing, we focused on transcripts for which one or more exons, but not the whole transcript, was differentially expressed. Genetrail2 analysis suggests that these transcripts may be associated with a variety of cellular processes that are not prominent in differentially expressed transcripts under these same conditions [32]. Prominent among the predicted pathways are processes involved in nuclear processes such as chromosomes and chromatin structure (**S10 Table**). Examples of differential exon usage in absence or presence of Strap are the transcription regulator *Max* (Myc associated factor X), the RNAse L inhibitor *Abce1* (ATP-binding cassette, sub-family E1) that is involved in gene transcription and mRNA translation and *Fdft1* (farnesyl diphosphate-farnesyl transferase 1) that has a role in cholesterol synthesis (**S5 Fig, panel B**). In case of Abce1 it seems that alternative transcription start site causes the alternative expression.

## Discussion

The RNA-binding protein Csde1 is essential for erythropoiesis and strongly upregulated in erythroblasts. Reduced expression is associated with DBA [5]. Csde1 binds a subset of transcripts that mainly encode proteins involved in protein homeostasis [15]. The effect of Csde1 on mRNA stability and protein expression of associated transcripts differed. Some were differentially expressed at the transcript level, others at the protein level, which suggested that the nature of the Csde1-containing protein complex is important for the effect of Csde1 on expression levels of associated transcripts and encoded proteins. We identified Strap as a protein that is abundantly associated with Csde1, whereas also Pabpc1 and Pabpc4 were enriched in Csde1 pull downs. Knockdown of Strap did not affect Csde1-mRNA interactions, but deregulated mRNA or protein expression of some Csde1-bound transcripts, including Pabpc1 and Pabpc4. Strap regulated expression of several transcripts involved in protein and mRNA homeostasis and had an effect on the expression of proteins involved in the hypoxia pathway including expression of Eif4g3, a scaffolding component of an alternate eIF4F active under hypoxic conditions.

The transcripts bound by Csde1 that were detected at a FDR <0.05 in virus transduced MEL cells only partially overlapped with the pool of transcripts that we previously detected in MEL WT cells [15].

The question remains why we detected a difference in Csde1 bound transcripts in transduced MEL cells compared to the previously described Csde1-bound transcripts in MEL WT cells [15]. We hypothesize that this may be due to the viral response. As part of the viral response, the expression of Csde1 is strongly increased ([15] and RNA expression data of this study). In general, transcripts that were detected as bound by Csde1 in MEL WT but not in transduced MEL cells were detected at lower level in the pull down from transduced MEL cells, and transcripts that were not detected in the pull down of MEL WT cells but that are detected in this study are detected at increased levels in transduced MEL cells (S1 Fig, panel B). However, this is not true for all transcripts. The virus response may alter transcript binding by Csde1 but this needs to be tested in a separate experimental design.

The modest changes in protein expression may be due to the fact that RNA and protein expression were analyzed 72 hours following transduction of shRNA expression vectors, which is when knock down starts to take effect. At this time, reduced expression of proteins with a long half-life may be underestimated.

At the RNA level, knockdown of Strap altered expression of Csde1-bound transcripts involved in translational control, proteasome functionality and cell cycle regulation. The overlap between transcripts involved in cell cycle control and proteasome functionality suggest that Strap may affect the cell cycle via post-translational (de)ubiquitination. Strap knockdown reduced expression of Ranbp1, which controls the cell cycle via nuclear transport of RNA and proteins [40,41]. Ranbp1 is highly expressed in erythroid progenitors relative to more primitive CD34+ progenitors. The *Ranbp1* transcript contains an uORF in its 5´UTR that controls *Ranbp1* translation in response to eIF2 phosphorylation induced by tunicamycin in erythroid cells [42,43]. Strap knockdown also reduced expression of Thoc5, Hmbs, and Ostf1. Thoc5 is a nuclear exporter of mRNAs essential for the maintenance of hematopoiesis [44]. Hmbs catalyzes a crucial step in heme biosynthesis and is oppositely regulated from Hif-1 under hypoxic conditions [36], and Ostf-1 is induced by hypoxia [37,45]. Regulation of these transcripts suggest a possible role for Strap in the regulation of terminal erythroid differentiation and the hypoxic response.

Also decreased after Strap knockdown is Vimentin (Vim), which is repressed during terminal erythroid differentiation [46–48]. Biotin pulldown in erythroblasts did not identify Vim as a Csde1-bound transcript [15]. However, an iCLIP approach demonstrated that Csde1 binds the 3’UTR of Vim in human melanoma cells [14]. Knockdown of Csde1 in these cells reduced expression of Vim via decreased ribosome occupancy in melanoma cells. Although the association between Vim and Csde1 is too weak in erythroblasts to be detected, Strap is necessary for both transcript and protein expression, suggesting an effect mainly at the transcriptional level.

Interestingly, we not only substantiated the previously observed interaction of Csde1 with Pabpc1 [11,17], but we also demonstrated interaction of Csde1 with Pabpc4. It is notable that our data did not identify other proteins that were previously shown to cooperate with Csde1, such as PTB [7,19], hnRNP C1/C2 [10] or 4E-T [28]. As Csde1 is essential for erythroblast proliferation and differentiation, we hypothesize that the interaction between Csde1, Strap and Pabpc1/4 may be of particular importance during erythropoiesis.

At protein level, loss of Strap resulted in increased Pabpc1 protein expression despite reduction of *Pabpc1* mRNA expression. Csde1 forms a complex with Pabpc1 and Imp1 (Imprintor 1) that binds an adenine-rich autoregulatory sequence (ARS) in the 5’UTR of *Pabpc1* and represses translation of *Pabpc1*. Binding of the trimeric UNR/Imp/Pabp complex inhibits *Pabpc1* translation which then relieves the repression in an autoregulatory loop [31]. We previously showed that reduced expression of Csde1 resulted in increased Pabpc1 protein expression from reduced levels of Pabpc1 mRNA, which is in agreement with its proposed role in the autoregulatory loop of Pabpc1 [15]. The findings that loss of Strap also results in increased Pabpc1 protein from reduced Pabpc1 mRNA, and the interaction of Strap with Csde1, suggests that Strap may be a functional part of the same Csde1/Pabpc1/Imp1 complex. Loss of Strap also increased the protein:mRNA ratio of Pabpc4. A similar autoregulatory loop involving Strap and Csde1 may exist for *Pabpc4* as it does for *Pabpc1*. The strongly reduced expression of *Pabpc4* mRNA nevertheless resulted in reduced Pabpc4 protein expression, while Pabpc1 protein is upregulated.

Loss of Strap also increased expression of the Pabpc4 antagonist Paip2 (not encoded by a Csde1-bound transcript). Concurrently, loss of Strap reduced expression of Eif4g3 expression. Pabpc4, bound to the poly(A) tail, associates with eIF4G, which is part of the pre-initiation scanning complex. This interaction increases mRNA stability and translation [49], and is negatively regulated by competitive binding of Paip2 to eIF4G [50]. Relevantly, eIF4g3 replaces eIF4g1 in an alternate, hypoxic eIF4F complex, which selectively promotes the translation of Hif target transcripts [35]. Loss of Strap reduces both eIF4G3 and Pabpc4, and enhances Paip2. Thus, Strap is required for gene expression regulation via eIF4G3/Pabpc4Pabpc4 is especially important in erythropoiesis. Pabpc4 stabilizes mRNA transcripts encoding shortened poly(A) tails, including *hα-globin*, *Hif1a*, and *Gata2* [33]. Pabpc4-depleted cells display elevated levels of *c-Kit*, *c-Myb*, *c-Myc*, *CD44*, and *Stat5a*, all genes that are repressed during terminal erythroid differentiation. Taken together we hypothesize that Strap may amplify Pabpc4-mediated translational regulation, possibly with a specific role under hypoxic conditions.

The length of the polyA tail determines how many Pabp molecules can bind to an mRNA transcript. Binding of Pabp protects the polyA tail from deadenylation, for instance by the Cnot1 (CCR4-Not1) deadenylation complex. Cnot1 is downregulated upon Strap knockdown at the protein level [51]. Cnot1 has distinct functions. It has been shown to indirectly interact with both Strap and Csde1 via 4-ET, an eIF4E shuttling protein that transports mRNAs between P-bodies and the cytoplasm [28]. Proteins binding to AU-rich elements in the 3´UTR of transcript can recruit the Cnot1 complex to de-adenylate the polyA tail which may lead to mRNA degradation. The mechanism by which Strap influences the expression of Cnot1 is unclear, but it can be postulated that loss of Strap and subsequent loss of Cnot1 disrupts mRNA degradation and silencing.

Although they are not encoded by Csde1-bound transcripts, Strap influences the expression of several other proteins involved in translational regulation and/or hematopoiesis, the most prominent of which is Elavl1 (HuR), a member of the Elav family of AU-binding protein with well-established roles in hematopoiesis [52]. Elavl1 binding to AU-rich elements protects transcripts from degradation by preventing the recruitment of Cnot1. For instance, Elavl1 binds to the 3’UTR of Gata1, stabilizing Gata1 translation [53]. Knockdown of Elav1a results in the disruption of embryonic erythropoiesis in zebrafish. Elavl1 also stabilizes the *Vegf-a* transcript, a Hif1a-inducible transcript that promotes angiogenesis under hypoxic conditions [54]. Strap knockdown reduced *Vegfa* expression at the RNA level. Importantly, Elavl1 regulates alternative splicing of *eIF4enif1*, the transcript that encodes 4E-T [55]. The absence of Elav1l promotes the expression of the shorter, more stable 4E-T isoform, resulting in drastically increased P-body formation and mRNA turnover of Hif1a, while suppressing angiogenesis via reduced expression of Vegfa. Another member of the Elav family specific to neurons, Elav4 (HuD), is colocalized with the SMN complex in neuronal cells, though this interaction does not depend on the interaction between HuD and RNA [56]. Strap is essential for the assembly of the SMN complex [27], and SMN deficiency is results in the decreased expression of HuD [56]. It is tempting to speculate that knockdown of Strap in erythroblasts results in a comparable loss of HuR via deregulation of the SMN complex.

In conclusion, Strap and Csde1 regulate expression of transcripts and encoded proteins essential for erythropoiesis. Strap does not affect which transcripts are bound by Csde1, but nevertheless alters expression of select Csde1-bound transcripts. These transcripts are involved in hypoxia and terminal erythroid differentiation.

## Materials and methods

### Cell culture

Murine erythroleukemia (MEL, mouse erythroblasts transformed with Friend virus, see references [57,58]) and HEK293T cells were cultured in RPMI, and DMEM respectively (Thermofisher), supplemented with 10% (vol/vol) fetal calf serum (FCS; Bodinco), glutamine and Pen-Strep (Thermofisher). MEL cells expressing BirA, or BirA plus biotag-Csde1 were described previously [5]. Cell number and size were determined using CASY cell counting technology (Roche).

### Lentivirus production and transductions

HEK293Ts were transfected with pLKO.1-puro lentiviral construct containing shRNA sequences for Strap: TRCN0000088837 and a scrambled control shRNA: SHC002 (MISSION TRC-Mm 1.0 shRNA library; Sigma-Aldrich; available on the BloodWeb site), pMD2.G, and pSPAX.2 packaging plasmids (gift of T. van Dijk, Erasmus MC, Rotterdam, The Netherlands) using 0.5M CaCl2 and HEPES (Thermofisher). 72 hours after transduction, viral supernatant was harvested and concentrated using 5% w/v PEG8000 (Sigma). MEL cells were transduced with a multiplicity of infection of 3–5 and addition of 8 μg/mL of Polybrene (Sigma-Aldrich). Transduced cells were selected with 1 μg/ml puromycin 24 hours after transduction.

### Protein-RNA and protein-protein pulldown for Csde1

Cell lysates, SDS-PAGE, and Western blotting were performed as described previously [5]. Biotagged Csde1 containing complexes were collected on streptavidine beads from 10^8^ MEL-BirA or MEL-BirA-Csde1-tag cells (3 biological replicates each) using a previously described protocol [59], with the following modifications. M-270 Dynabeads (Thermofisher; 100μl per 10^8^ cells) were blocked for 1 hour at 4°C in 5% chicken egg albumin and then washed 3x in ice-cold NT2 buffer [50mM Tris-HCl (Sigma-Aldrich), 150mM NaCl (Sigma-Aldrich), 1mM MgCl_2_ (Thermofisher) and 0.05% NP40 (Sigma-Aldrich)]. Cells were lysed in 850μl cold NT2, supplemented by 200U RNAse Out (EMD Bioscience), 400μM vanadyl ribonucleoside complexes (VRC, New England Biolabs) and 20mM EDTA (EM Science), and incubated with the beads for 2 hours at 4°C. Beads were immobilized in a magnet rack, washed 5x with NT2 containing 0.3M NaCl, split into a protein and an RNA fraction. The protein fraction was eluted via boiling in 1x Laemmli buffer (Sigma-Aldrich) for 5 minutes. RNA fractions were purified using Trizol (Invitrogen), precipitated in isopropanol and washed in 75% ethanol.

### SDS-PAGE, Western blotting and silver staining

MEL cells were fractionated into cytoplasmic and nuclear components using a Cell Fractionation Kit—Standard (ab109719, Abcam), or total MEL cell protein lysates were generated. Proteins were detected via SDS-PAGE and Western blotting as described (Horos et al., 2012). Antibodies used were directed against Strap (sc-136083, Santa Cruz), Csde1 (NBP1-71915, Novus Biological), Stat5 (sc-835, Santa Cruz), Lamin B1 (ab133741, Abcam) and alpha Tubulin (ab4074, Abcam). Fluorescently labeled secondary antibodies for visualization with Odyssey were IRDye 680RD Donkey anti-Rabbit IgG (926–68073, Licor) and IRDye 800CW Donkey anti-Mouse IgG (925–32212, Licor), or using the Pierce enhanced chemiluminescence (ECL) kit (Thermofisher). Silver staining was performed using a SilverQuest™ Silver Staining Kit (LC6070, Thermofisher).

### Mass spectrometry

Eluted peptides were processed as described by [60]. Samples were subjected to mass spectrometry using label-free quantification. All data was analyzed and processed with MaxQuant for peptide identification and quantification [61]. Downstream statistical analysis was performed with Perseus v1.5.1.6 [62]. All peptides matching the reverse database, potential contaminants, and those only identified by site were filtered out. To be considered for analysis, a peptide had to be detectable within all triplicates of at least one clone. Prior to statistical testing, peptide counts were 2log transformed. Because failures to detect a given peptide is sometimes due to insufficient depth, missing values were imputed from the normal distribution with a width of 0.3 and a downshift of 1.8. These values were later de-imputed prior to visualization and production of the final tables. For two-way comparisons between groups, a *t*-test applying an artificial within groups variance of S0 = 0.8 was used [31]. For all analyses, a Benjamini-Hochberg false discovery rate of < 0.05 was applied. The mass spectrometry proteomics data have been deposited in the ProteomeXchange Consortium via the PRIDE partner repository with the dataset identifier PXD006358 (https://www.ebi.ac.uk/pride/).

### RNA-sequencing

RNA-seq on Csde1-associated transcripts after Strap knockdown was performed by the Leiden Genome Technology Center (LGTC, Leiden), using library preparation following the template-switch protocol (Clontech), and Nextera tagmentation. Samples were split across three MiSeq (Illumina) lanes (2x75bp, paired end). RNA expression by total mRNA sequencing after Strap knockdown was performed by Novogene Co., LTD. on mRNA enriched on oligo(dT) beads. RNA was randomly fragmented, and processed with the NEB Next® Ultra™ RNA Library Prep Kit using random hexamers. The library was sequenced using Illumina HiSeq 2500 (2x150bp, paired end). Sequence quality for both experiments was checked using Fastqc (Babraham Bioinformatics).

Spliced Transcripts Alignment to a Reference (STAR, [63]) was used to align the sequences to the mouse mm10 genomic reference sequence, using the following parameters—outFilterMultimapNmax 20,—outFilterMismatchNmax 1,—outSAMmultNmax 1,—outSAMtype BAM SortedByCoordinates, quantMode GeneCounts, -outWigType wiggle, -outWigStrand Stranded,—outWigNorm RPM. A gtf file accessed from the UCSC genome browser on 11-Sept-2015 was passed to STAR using–sjdbGTFfile. The read count tables were subjected to differential expression analysis with DESeq2 [64]. DESeq2 implements a negative binomial generalized linear model to identify differential expressed/enriched transcripts. This method normalizes raw counts by adjusting for a size factor to account for discrepancies in sequencing depth between samples. The normalized counts are subsequently subjected to a Wald test with a Benjamini-Hochberg correction for multiple testing (FDR, false discovery rate). In pulldown experiments, transcripts were filtered for a positive fold change of Csde1-tag vs BirA. For simple pairwise comparisons in RNAseq or RIPseq (for example: Sc Csde1-tag vs Sc BirA in a pulldown, or KD Strap vs Sc for total RNAseq), the formula ~replicate + condition was used. When determining whether knockdown of Strap influences which transcripts are bound to Csde1, the following interaction model was applied: ~ replicate + shRNA + pulldown + shRNA:pulldown, where shRNA indicates treatment with anti-Strap or control shRNA, replicate indicates the batch, and pulldown indicates the presence or absence of biotagged Csde1. DESeq2 also provides a function for principal component analysis (PCA). Additional visualizations were made using R packages ggplots and pheatmap. Overrepresentation Analysis (ORA) and Gene Set Enrichment Analysis (GSEA) for GO-terms and pathways was performed on significant transcripts with GeneTrail2 [32]. Original sequencing results have been deposited in the BioProject Database under project ID PRJNA379114 (https://www.ncbi.nlm.nih.gov/bioproject/).

For analysis of differential exon usage, we used the DEXSeq package [38,39]. DEXSeq uses a similar method as DESeq2, except that counts for each exon are included in the model alongside counts for the total transcript. A likelihood ratio test is then performed between a model that includes an exon:condition interaction term with one that does not. The result is the identification of differential expression of exons within a transcript, accounting for changes in total transcript expression due to Strap knockdown.

### Correlation of RNA and protein expression levels

RNA expression levels were normalized as reads per kilobase of transcript per million mapped reads (RPKM). In mass spectrometry, iBAQ values (as determined via MaxQuant) were normalized via a scaling factor calculated by dividing the sum of intensities from each sample by the intensity sum of a reference sample. A Spearman rank correlation coefficient was calculated between 10log(RPKM) and 10log(iBAQ).

## Supporting information

S1 Fig Detection level of transcripts in pull down of biotagged Csde1 on strepatavidine beads. MEL cells expressing biotin ligase BirA with and without biotagged Csde1 (MEL WT) were treated with anti-Strap (Sh Strap) and control (Sc Strap) shRNA. They were then subjected to a protein-RNA pulldown on streptavidin beads followed by RNA sequencing. Shown are the reads from pull downs of cells that express biotagged Csde1. The base mean is the mean (normalised) read count in counts per million (cpm) from 3 independent experiments. MEL WT cells were previously analysed (ref. 15). Reads from MEL WT were normalised apart from the virus transdusced MEL cells (sh Strap and Sc Strap) **(A)** A comparison between the base mean of pull downs from cells treated with shRNA against Strap and the pull down of cells treated with control virus (Sc Strap) **(B)** A comparison between the base mean of pull downs from MEL WT cells and the pull down of cells treated with control virus (Sc Strap). Colours indicate whether transcripts were specifically pulled down from cells expressing biotagged Csde1 versus BirA only at a FDR < 0.05. Red dots detected in parental MEL and in shRNA treated MEL; blue dots detected in previous study with parental MEL; green dots detected in shRNA treated MEL. Many transcripts are detected in both experiments (red dots). The transcripts that were not detected in the current study with virus transduced cells are detected at lower levels in the pull down of MEL WT cells, and transcripts that are detected in the current study but not in MEL WT are detected at higher levels upon virus transduction. An increased or decreased detection level does not affect the fold-change increase in cells that do or do not express biotaged Csde1. We assume that this is due to overall expression level.
(PDF)

S2 Fig MA plot of the Csde1 RIPseq interaction model. MEL cells expressing biotin ligase BirA with and without biotagged Csde1 were treated with anti-Strap and control (Sc) shRNA. They were then subjected to a protein-RNA pulldown followed by RNA sequencing. Cells expressing BirA without biotagged Csde1 represent pulldown background. An interaction term was used to model the effect of Strap knockdown on Csde1 transcript affinity. Significant transcripts are highlighted in red.
(PDF)

S3 Fig Principal component analysis on RNAseq results of Strap knockdown in MEL. Depicted are both shRNA and replicate groups, indicating that the shRNA is responsible for the majority of variation between samples. PC2 (12%) is the result of minor batch effects.
(PDF)

S4 Fig Reduction of Csde1 expression does not alter Strap localization. **(A)** Total cell lysates of MEL cells expressing BirA plus or minus biotagged Csde1 was used to pull down Csde1 using streptavidin beads. lysates were loaded on SDS-PAGE. Western blots were probed with anti-Csde1 and anti-Strap antibodies. The tagged Csde1 protein pulled down on streptavidin beads, has been extend with 23 amino acids (masslrqildsqkmewrsnaggs; Csde1 itself is ~90kD, 767 aa, size increase of tagged protein is <3%) **(B)** Western blot loaded with lysate fractions from parental MEL cells (**WT**), or CRISPR clones with bi-allelic deletions in Csde1 indicated as hypomorphic (**hypomorph**, in-frame deletion of the 1^st^ cold shock domain), or deleted (**HOM KO**, out-of-frame deletion of the 1^st^ cold shock domain, unexpectedly resulting in low expression of a N-terminally truncated protein) and heterozygous deletion (**HET KO**). Lysates (T, total lysate) were fractionated into cytoplasmic C) and nuclear (N) extracts. Numbers identify specific CRISPR clones (see ref. 15) Antibody staining was performed for Csde1, Strap, Lamin B1 (nuclear control), and Tubulin (cytoplasmic control). Strap but not Csde1 is partly present in the nucleus. In this experiment the nuclear expression of Strap was only detected upon prolonged exposure.
(PDF)

S5 Fig Differentially expressed exons. **(A)** Venn diagram depicting the number of Csde1-bound transcripts (blue), and the number of differentially expressed transcripts detected at the transcript level (orange) or single exon level (brown; number of exons between parenthesis) comparing MEL cells treated with Sc control shRNA or anti-Strap. (**B**) Examples of transcripts with alternative exon usage between MEL cells expressing Sc (blue line) or anti-Strap (red line) shRNA. Transcript names (short and full) and function are indicated, expression is in cpm on a 10log scale. Exons are numbered on the x-axes, which corresponds to the graphic representation of all exons (in grey) below, together with known transcript variants. The differentially expressed exon is pink, and indicated with a red arrow.
(PDF)

S1 Table A comparison of results between Csde1 pulldowns, in untransduced MEL, MEL transduced with control shRNA (Sc002), and MEL transduced with anti-Strap shRNA. (XLSX)

S2 Table Interaction-term significant transcripts in Csde1 RIPseq with Strap knockdown. (XLSX)

S3 Table Overrepresentation analysis of all Csde1-associated transcripts in S1 Table. (XLSX)

S4 Table Significant transcripts in total mRNA sequencing after Strap knockdown. (XLSX)

S5 Table Significant proteins after KD Strap vs Sc. (XLSX)

S6 Table Gene set enrichment analysis of significant transcripts (total mRNA) after Strap knockdown. (XLSX)

S7 Table Overrepresentation analysis of significant proteins after KD Strap vs Sc. (XLSX)

S8 Table Genewise Spearman correlation between RPKM and iBAQ after Strap knockdown. (XLSX)

S9 Table Differential exon usage analysis after Strap knockdown. (XLSX)

S10 Table Overrepresentation analysis of transcripts with the differential exon usage after Strap knockdown. (XLSX)
