# Supplementary material for: Strap associates with Csde1 and affects expression of select Csde1-bound transcripts
Source: PLoS One. 2018 Aug 23;13(8):e0201690. doi: 10.1371/journal.pone.0201690 (PMC6107111; doi:10.1371/journal.pone.0201690)
Supplement: S1 Fig — MEL cells expressing biotin ligase BirA with and without biotagged Csde1 (MEL WT) were treated with anti-Strap (Sh Strap) and control (Sc Strap) shRNA. They were then subjected to a protein-RNA pulldown on streptavidin beads followed by RNA sequencing. Shown are the reads from pull downs of cells that express biotagged Csde1. The base mean is the mean (normalised) read count in counts per million (cpm) from 3 independent experiments. MEL WT cells were previously analysed (ref. 15). Reads from MEL WT were normalised apart from the virus transdusced MEL cells (sh Strap and Sc Strap) (A) A comparison between the base mean of pull downs from cells treated with shRNA against Strap and the pull down of cells treated with control virus (Sc Strap) (B) A comparison between the base mean of pull downs from MEL WT cells and the pull down of cells treated with control virus (Sc Strap). Colours indicate whether transcripts were specifically pulled down from cells expressing biotagged Csde1 versus BirA only at a FDR < 0.05. Red dots detected in parental MEL and in shRNA treated MEL; blue dots detected in previous study with parental MEL; green dots detected in shRNA treated MEL. Many transcripts are detected in both experiments (red dots). The transcripts that were not detected in the current study with virus transduced cells are detected at lower levels in the pull down of MEL WT cells, and transcripts that are detected in the current study but not in MEL WT are detected at higher levels upon virus transduction. An increased or decreased detection level does not affect the fold-change increase in cells that do or do not express biotaged Csde1. We assume that this is due to overall expression level. (PDF) [file pone.0201690.s001.pdf]

S1 Figure

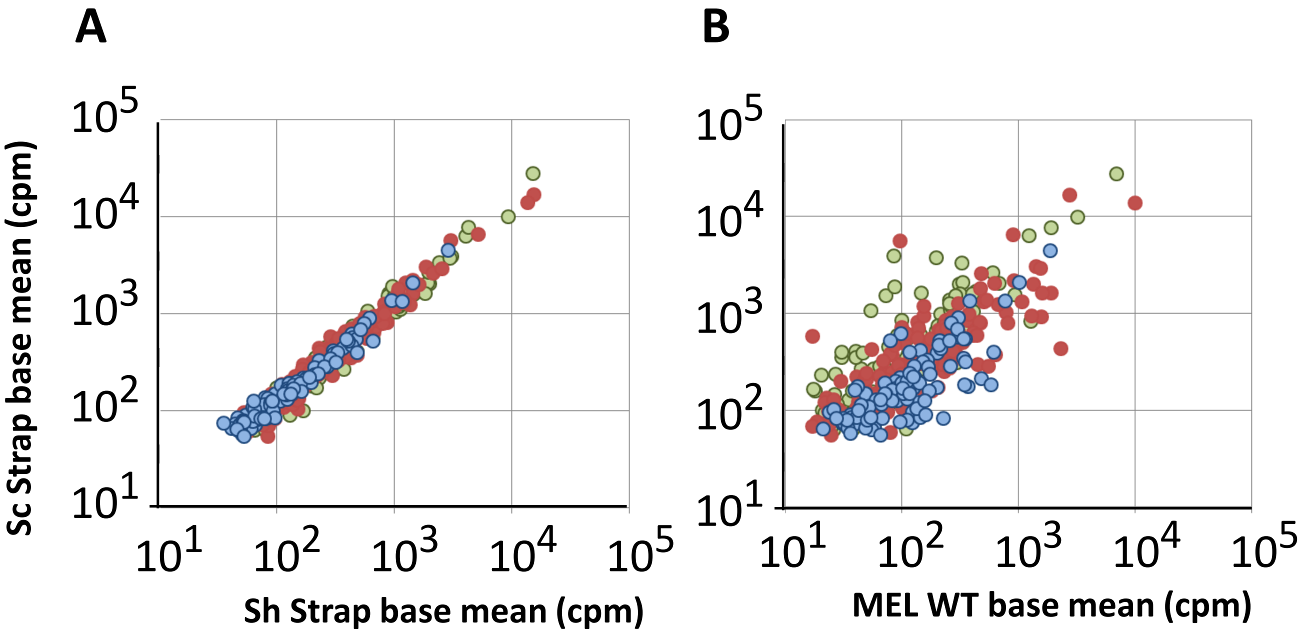

S1 Figure. Detection level of transcripts in pull down of biotagged Csde1 on strepatavidine beads. MEL cells expressing biotin ligase BirA with and without biotagged Csde1 (MEL WT) were treated with anti-Strap (Sh Strap) and control (Sc Strap) shRNA. They were then subjected to a protein-RNA pulldown on streptavidin beads followed by RNA sequencing. Shown are the reads from pull downs of cells that express biotagged Csde1. The base mean is the mean (normalised) read count in counts per million (cpm) from 3 independent experiments. MEL WT cells were previously analysed (ref. 15). Reads from MEL WT were normalised apart from the virus transdusced MEL cells (sh Strap and Sc Strap) (**A**) A comparison between the base mean of pull downs from cells treated with shRNA against Strap and the pull down of cells treated with control virus (Sc Strap) (**B**) A comparison between the base mean of pull downs from MEL WT cells and the pull down of cells treated with control virus (Sc Strap). Colours indicate whether transcripts were specifically pulled down from cells expressing biotagged Csde1 versus BirA only at a FDR < 0.05. Red dots detected in parental MEL and in shRNA treated MEL; blue dots detected in previous study with parental MEL; green dots detected in shRNA treated MEL. Many transcripts are detected in both experiments (red dots). The transcripts that were not detected in the current study with virus transduced cells are detected at lower levels in the pull down of MEL WT cells, and transcripts that are detected in the current study but not in MEL WT are detected at higher levels upon virus transduction. An increased or decreased detection level does not affect the fold-change increase in cells that do or do not express biotaged Csde1. We assume that this is due to overall expression level
