# Supplementary material for: Strap associates with Csde1 and affects expression of select Csde1-bound transcripts
Source: PLoS One. 2018 Aug 23;13(8):e0201690. doi: 10.1371/journal.pone.0201690 (PMC6107111; doi:10.1371/journal.pone.0201690)
Supplement: S2 Fig — MEL cells expressing biotin ligase BirA with and without biotagged Csde1 were treated with anti-Strap and control (Sc) shRNA. They were then subjected to a protein-RNA pulldown followed by RNA sequencing. Cells expressing BirA without biotagged Csde1 represent pulldown background. An interaction term was used to model the effect of Strap knockdown on Csde1 transcript affinity. Significant transcripts are highlighted in red. (PDF) [file pone.0201690.s002.pdf]

S2 Figure

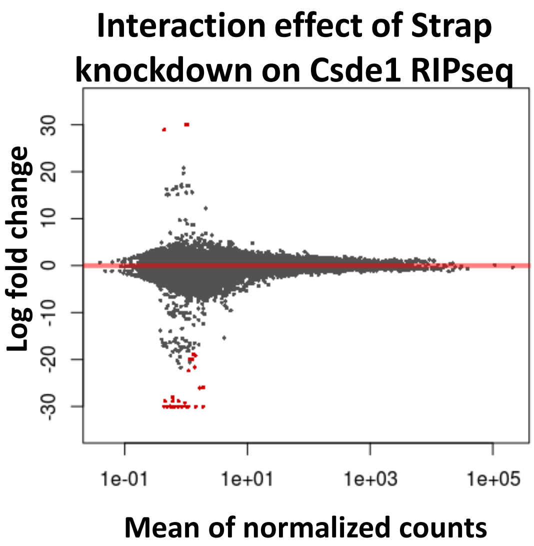

S2 Figure. MA plot of the Csde1 RIPseq interaction model. MEL cells expressing biotin ligase BirA with and without biotagged Csde1 were treated with anti-Strap and control (Sc) shRNA. They were then subjected to a protein-RNA pulldown followed by RNA sequencing. Cells expressing BirA without biotagged Csde1 represent pulldown background. An interaction term was used to model the effect of Strap knockdown on Csde1 transcript affinity. Significant transcripts are highlighted in red.
