# Supplementary material for: Strap associates with Csde1 and affects expression of select Csde1-bound transcripts
Source: PLoS One. 2018 Aug 23;13(8):e0201690. doi: 10.1371/journal.pone.0201690 (PMC6107111; doi:10.1371/journal.pone.0201690)
Supplement: S3 Fig — Depicted are both shRNA and replicate groups, indicating that the shRNA is responsible for the majority of variation between samples. PC2 (12%) is the result of minor batch effects. (PDF) [file pone.0201690.s003.pdf]

S3 Figure

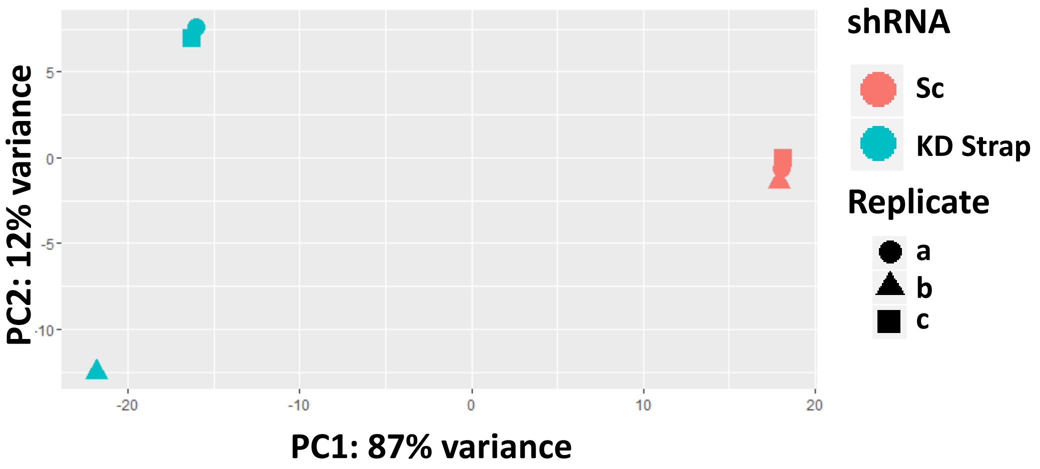

S3 Figure. Principal component analysis on RNAseq results of Strap knockdown in MEL. Depicted are both shRNA and replicate groups, indicating that the shRNA is responsible for the majority of variation between samples. PC2 (12%) is the result of minor batch effects.
