# Supplementary material for: Strap associates with Csde1 and affects expression of select Csde1-bound transcripts
Source: PLoS One. 2018 Aug 23;13(8):e0201690. doi: 10.1371/journal.pone.0201690 (PMC6107111; doi:10.1371/journal.pone.0201690)
Supplement: S4 Fig — (A) Total cell lysates of MEL cells expressing BirA plus or minus biotagged Csde1 was used to pull down Csde1 using streptavidin beads. lysates were loaded on SDS-PAGE. Western blots were probed with anti-Csde1 and anti-Strap antibodies. The tagged Csde1 protein pulled down on streptavidin beads, has been extend with 23 amino acids (masslrqildsqkmewrsnaggs; Csde1 itself is ~90kD, 767 aa, size increase of tagged protein is <3%) (B) Western blot loaded with lysate fractions from parental MEL cells (WT), or CRISPR clones with bi-allelic deletions in Csde1 indicated as hypomorphic (hypomorph, in-frame deletion of the 1st cold shock domain), or deleted (HOM KO, out-of-frame deletion of the 1st cold shock domain, unexpectedly resulting in low expression of a N-terminally truncated protein) and heterozygous deletion (HET KO). Lysates (T, total lysate) were fractionated into cytoplasmic C) and nuclear (N) extracts. Numbers identify specific CRISPR clones (see ref. 15) Antibody staining was performed for Csde1, Strap, Lamin B1 (nuclear control), and Tubulin (cytoplasmic control). Strap but not Csde1 is partly present in the nucleus. In this experiment the nuclear expression of Strap was only detected upon prolonged exposure. (PDF) [file pone.0201690.s004.pdf]

S4 Figure

A

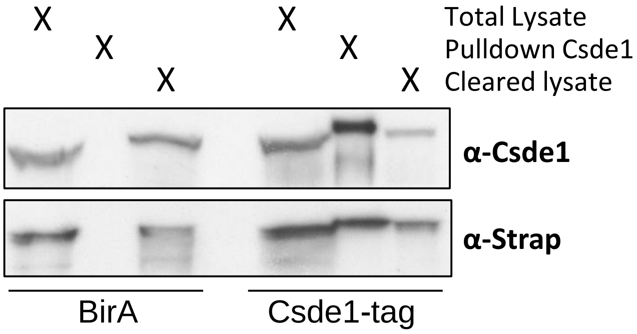

B

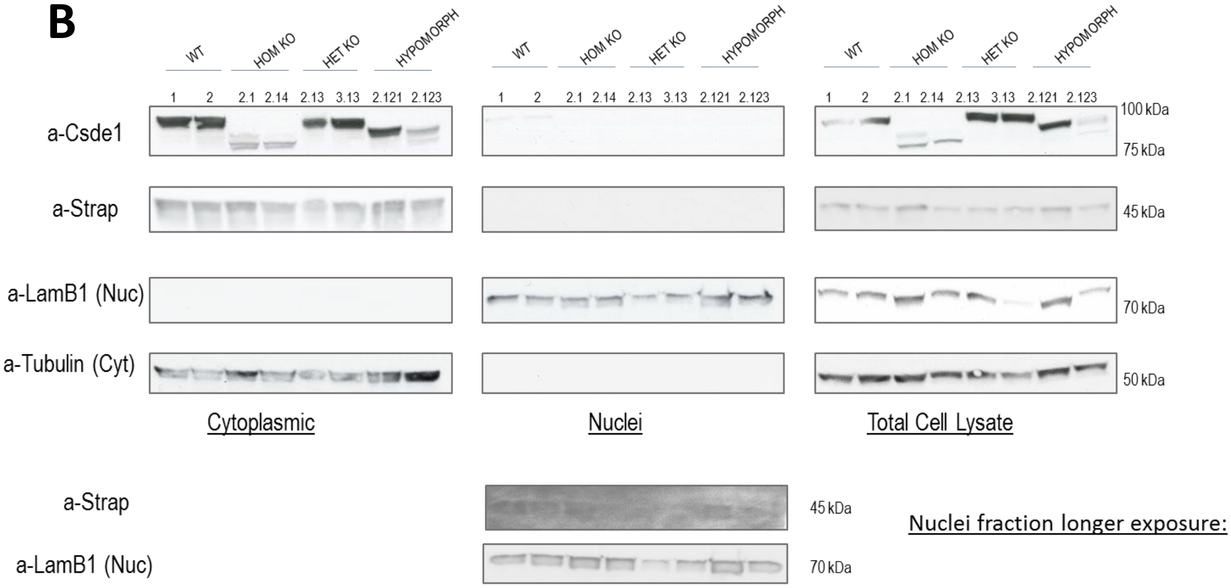

S4 Figure. Reduction of Csde1 expression does not alter Strap localization. (A) Total cell lysates of MEL cells expressing BirA plus or minus biotagged Csde1 was used to pull down Csde1 using streptavidin beads. lysates were loaded on SDS-PAGE. Western blots were probed with anti-Csde1 and anti-Strap antibodies. The tagged Csde1 protein pulled down on streptavidin beads, has been extend with 23 amino acids (MASSLRQILDSQKMEWRSNAGGS; Csde1 itself is ~90kD, 767 aa, size increase of tagged protein is <3%) **(B)** Western blot loaded with lysate fractions from parental MEL cells (**WT**), or CRISPR clones with bi-allelic deletions in Csde1 indicated as hypomorphic (**hypomorph**, in-frame deletion of the 1st cold shock domain), or deleted (**HOM KO**, out-of-frame deletion of the 1st cold shock domain, unexpectedly resulting in low expression of a N-terminally truncated protein) and heterozygous deletion (**HET KO**). Lysates (T, total lysate) were fractionated into cytoplasmic C) and nuclear (N) extracts. Numbers identify specific CRISPR clones (see ref. 15) Antibody staining was performed for Csde1, Strap, Lamin B1 (nuclear control), and Tubulin (cytoplasmic control). Strap but not Csde1 is partly present in the nucleus. In this experiment the nuclear expression of Strap was only detected upon prolonged exposure
