# Supplementary material for: Strap associates with Csde1 and affects expression of select Csde1-bound transcripts
Source: PLoS One. 2018 Aug 23;13(8):e0201690. doi: 10.1371/journal.pone.0201690 (PMC6107111; doi:10.1371/journal.pone.0201690)
Supplement: S5 Fig — (A) Venn diagram depicting the number of Csde1-bound transcripts (blue), and the number of differentially expressed transcripts detected at the transcript level (orange) or single exon level (brown; number of exons between parenthesis) comparing MEL cells treated with Sc control shRNA or anti-Strap. (B) Examples of transcripts with alternative exon usage between MEL cells expressing Sc (blue line) or anti-Strap (red line) shRNA. Transcript names (short and full) and function are indicated, expression is in cpm on a 10log scale. Exons are numbered on the x-axes, which corresponds to the graphic representation of all exons (in grey) below, together with known transcript variants. The differentially expressed exon is pink, and indicated with a red arrow. (PDF) [file pone.0201690.s005.pdf]

S5 Figure

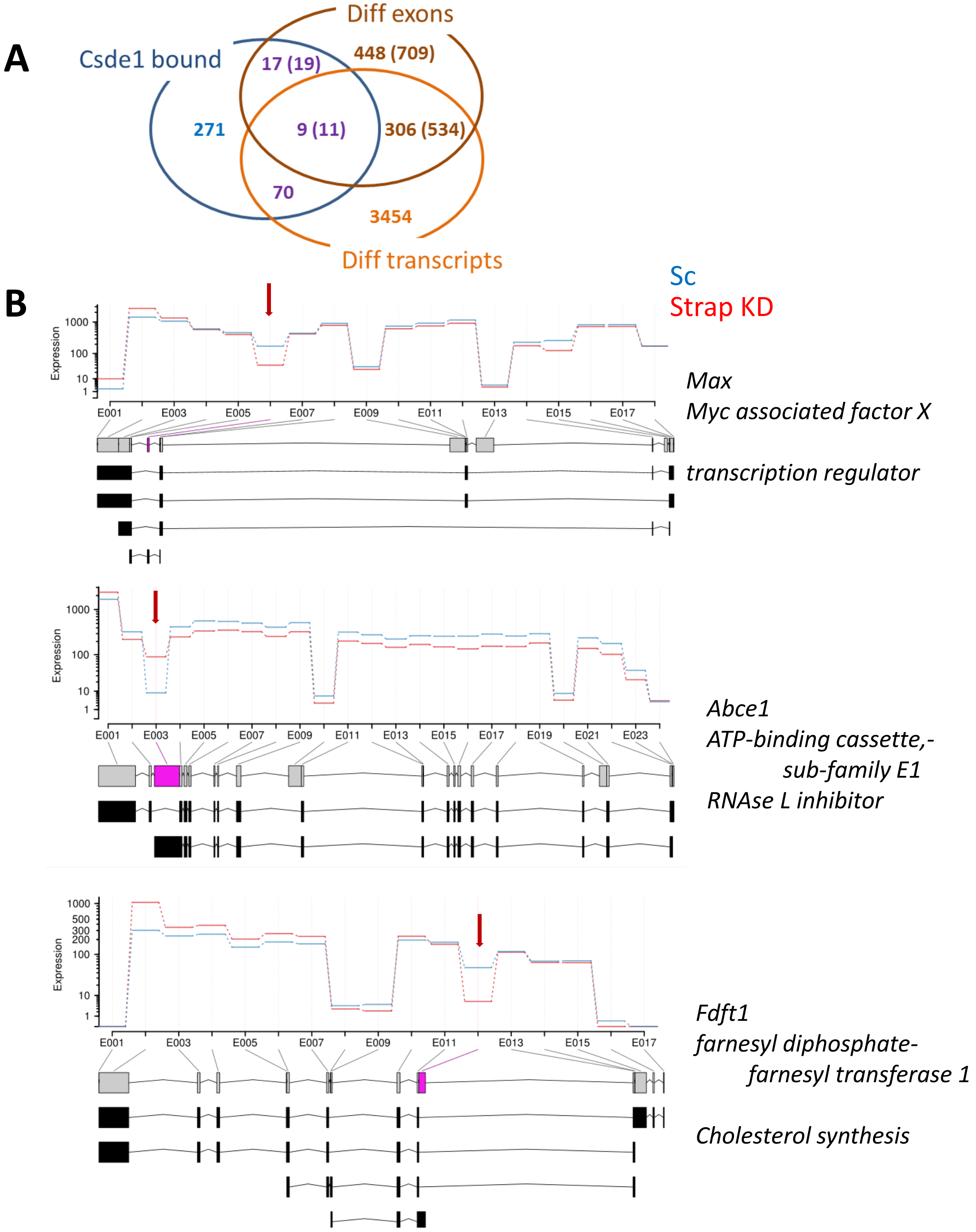

S5 Figure, Differentially expressed exons. (A) Venn diagram depicting the number of Csde1-bound transcripts (blue), and the number of differentially expressed transcripts detected at the transcript level (orange) or single exon level (brown; number of exons between parenthesis) comparing MEL cells treated with Sc control shRNA or anti-Strap. (**B**) Examples of transcripts with alternative exon usage between MEL cells expressing Sc (blue line) or anti-Strap (red line) shRNA. Transcript names (short and full) and function are indicated, expression is in cpm on a 10log scale. Exons are numbered on the x-axes, which corresponds to the graphic representation of all exons (in grey) below, together with known transcript variants. The differentially expressed exon is pink, and indicated with a red arrow
